# Dermatologists' Experience in Managing Body Dysmorphic Disorder in Saudi Arabia

**DOI:** 10.7759/cureus.80632

**Published:** 2025-03-15

**Authors:** Mohammed A AlFada

**Affiliations:** 1 Department of Dermatology, King Saud University, Riyadh, SAU

**Keywords:** body dysmorphic disorder, dermatologist, dermatology, dysmorphophobia, saudi arabia

## Abstract

Background: Preoccupation with a perceived minor or non-existent flaw in physical appearance that negatively impacts a patient’s quality of life is the cardinal feature of body dysmorphic disorder (BDD).

Objective: This study aims too explore how commonly dermatologists in Saudi Arabia (SA) encounter BDD patients and their preferred management approach.

Methods: We conducted a cross-sectional study using a previously validated questionnaire that included information about participants' demographics, the frequency of exposure to BDD patients, and their management approach. We used a purposeful sampling technique, distributing the survey via Google Forms to 127 dermatologists living in SA, regardless of their age, gender, position, clinical setting, or work experience, over a period of one month.

Results: A total of 118 (93%) participants had encountered at least one BDD patient at some point throughout their working life. Two-thirds of participants are currently treating BDD patients. Seventy-eight participants (62%) consider patient reassurance and condition explanation as their primary approach to managing BDD patients, despite knowing that antidepressants are considered the first-line treatment.

Conclusion: Most dermatologists in SA are aware of BDD and encounter it in their daily practice. While they recognize that antidepressants are the first-line treatment and that early involvement of a psychiatrist is paramount, most prefer to simply explain the nature of the condition to the patient and provide reassurance.

## Introduction

According to the Diagnostic and Statistical Manual of Mental Disorders, Fifth Edition (DSM-5) criteria, body dysmorphic disorder (BDD) is a psychiatric disorder that is characterized by preoccupation with a perceived flaw in physical appearance. Even if a person has a minor physical abnormality, the associated preoccupation and concern are usually very exaggerated. This obsession is usually persistent and causes significant distress for patients, often with a significant impact on their psychological, social, and occupational life. It is usually accompanied by repetitive physical behaviors such as mirror checking, excessive grooming, skin picking, reassurance-seeking, or mental acts such as comparing one’s appearance with that of others, muscle dysmorphia, and poor insight regarding these BDD beliefs [1].

BDD can affect any age group, but it usually starts in early adolescence. The average age of onset of BDD is 16.9 years. It has been reported that there is an inverse relationship between age and the prevalence of BDD, with about 80% of BDD cases manifesting before the age of 30. Some studies have shown a high female-to-male ratio of about 3:1, while others have shown equal gender distribution of the disorder. Furthermore, BDD has been associated with other mental disorders such as major depression, borderline personality disorder, eating disorders, and obsessive-compulsive disorder, as shown in a recent systematic review [2].

Patients with BDD are dissatisfied with their bodies and focus on non-existent or negligible skin anomalies such as small scars or mild acne, as well as features like hair density, nose shape, or skin color. Regardless of their particular focus, patients perceive themselves as ugly and unattractive. These preoccupations are difficult to control and persist for a long period (on average three to eight hours per day). Along with having to cope with these thoughts, BDD patients are usually engaged in compulsive behaviors to hide their perceived defects, such as checking themselves in mirrors, constantly grooming themselves, drawing comparisons between themselves and others, as well as using clothes, makeup, hairstyles, and body posture to camouflage the imperfections [3].

Although BDD is not a life-threatening condition, it does carry a number of risks for sufferers. First, around 30% of BDD patients manipulate their skin in various ways to improve its appearance. This sometimes includes using sharp objects like razors and needles, which can cause significant disfigurement or even life-threatening situations. Second, BDD is strongly associated with depression and anxiety, and the presence of these affective disorders can mask the symptoms of BDD and make it difficult to recognize. Of the BDD patients studied by Phillips et al., about 80% had suicidal ideation, and around one in four had attempted suicide [4,5]. Finally, BDD significantly affects the quality of patients’ social and professional lives to a degree comparable to having had a myocardial infarction (heart attack), severe depression, or type II diabetes. In extreme cases, it can result in unemployment and social isolation [6,7].

Given its profound psychological impact, BDD patients frequently seek treatment from non-psychiatric physicians, particularly dermatologists and plastic surgeons. However, limited research exists on the prevalence of BDD in dermatology settings in Saudi Arabia (SA) and how dermatologists manage such cases. A review of the literature identified only one study assessing the awareness and attitudes of general cosmetic treatment providers toward BDD in SA. Therefore, this study aims to investigate the frequency with which dermatologists in SA encounter BDD patients and their preferred management strategies.

## Materials and methods

This observational cross-sectional study was conducted between June 1 and June 30, 2022, using a specially designed questionnaire. The study included all dermatologists practicing in medical, cosmetic, or combined settings, either in governmental hospitals or private clinics, in three major SA cities, Riyadh, Jeddah, and Dammam, without exclusion criteria. We used a purposive sampling technique and estimated the sample size based on Charan and Biswas’s previously published review describing appropriate methods of sample size calculation [8]. As reported in a previous study, the expected proportion in the population was about 91% (expected proportion of physicians who encounter BDD patients) [9]. Considering that our confidence interval (CI) was 95% and the margin of error was 5%, the estimated sample size was 126 participants. The design of the questionnaire was previously described and validated by Szepietowski et al. [10] in their study evaluating Polish dermatologists’ experience with BDD patients. It was pretested through a small pilot before being distributed to participants.

The survey contained questions and statements divided into three sections. The first section focused on participants' demographics, such as age, gender, nationality, and work-related characteristics, including position, work experience, and clinical setting. The second section evaluated participants' exposure to BDD patients in practice within the current year, over the last five years, and throughout their entire working life. The third section assessed dermatologists’ approaches toward BDD patients and which pharmacological interventions they consider first-line if they chose to treat these patients. The survey was distributed to participants via Google Forms. Informed consent was obtained from all participants, and study approval was granted by the Institutional Review Board at King Saud University, Riyadh, SA (E-22-6842).

To evaluate any association between dermatologists' demographics, work-related characteristics, and their exposure to BDD patients, we categorized dermatologists into two groups: "ever encountered a BDD patient" and "never encountered a BDD patient." Second, their management approach was categorized as either passive management (reassurance and explanation) or active management (personal initiation of pharmacotherapy, referral to a psychiatrist, or treatment according to a psychiatrist’s advice). This allowed us to investigate any association between the aforementioned characteristics and the type of management approach toward BDD patients.

Categorical variables were described as frequencies, and univariate analysis was conducted using the chi-square test to explore associations between sociodemographic and work-related characteristics of participating dermatologists, their exposure to BDD patients, and their type of management approach. Only categorical variables with a P-value < 0.05 were submitted to multivariate binary logistic regression analysis. We opted to use binary logistic regression analysis because our dependent variables ("ever encountered a BDD patient" vs. "never encountered a BDD patient" and "active management" vs. "reassurance and explanation") were binary. The odds ratios (ORs) and their 95% CIs were calculated. All statistical tests were two-tailed, with a significance level of 0.05. All analyses were performed using IBM SPSS Statistics for Windows, Version 21 (Released 2012; IBM Corp., Armonk, New York).

## Results

Sample characteristics

A total of 127 dermatologists completed the survey. Of them, 67 (53%) were female and 82 (64.6%) were aged between 30 and 50 years. Almost all were Saudi nationals (120, 94%), and 73 (58%) worked exclusively in government institutes. Ninety-seven participants (76.4%) were consultants, and 86 (68%) had more than five years of experience in the field of dermatology (Table 1).

**Table 1:** Sociodemographic and work-related characteristics of study participants

Characteristics	Number (N=127)	Frequency (100%)
Age		
20-30	29	23%
31-40	65	51%
41-50	17	13%
51-60	12	10%
>60	4	3%
Gender		
Male	60	47%
Female	67	53%
Nationality		
Saudi	120	94%
Non-Saudi	7	6%
Clinical setting		
Governmental	73	58%
Private	12	10%
Mixed	41	32%
Professional position		
Consultant	97	76%
Resident	30	24%
Work experience		
1 year	3	2%
1-5 years	38	30%
6-10 years	44	35%
11-20 years	28	22%
21-50 years	14	11%

Prevalence of BDD cases encountered

Only about nine participants (7%) had never observed a single case of BDD during their entire time working in dermatology. On the other hand, 118 participants (93%) had encountered at least one patient suffering from BDD. In the last five years, 44 participants (34%) had seen one or two cases, 40 (31%) had seen three to five cases, and around 32 (26%) had encountered more than six cases of BDD. Eighty-one participants (64%) had seen patients suffering from BDD in the current year.

Dermatologists preferred management approach

In terms of treating patients with BDD, around 78 (61%) participants felt that it was enough to explain the nature of the condition to the patient and provide reassurance. However, 48 participants (38%) reported that they would involve a psychiatrist in their patient’s treatment, and about 43 (34%) would formally refer their patient to a psychiatrist. Only one participant (1%) planned to use pharmacological therapy to treat a patient suffering from BDD.

When asked about the first-line treatment for patients suffering from BDD, should they plan to treat them, 22 participants (55%) reported that they would use antidepressant medication. A total of 12 participants (30%) reported that they would use a placebo as the initial therapy. Around six participants (7.5%) would use either antipsychotic or anti-anxiety medication as their first-line therapy (Table 2).

**Table 2:** Dermatologists' exposure and management approach toward BDD patients BDD: body dysmorphic disorder.

Statement	Number (N=127)	Frequency (100%)
BDD cases observed in the whole working experience		
None	9	7%
1-2	29	23%
3-5	42	33%
6-10	11	9%
More than 10	36	28%
BDD cases observed in the last 5 years		
None	11	9%
1-2	44	34%
3-5	40	31%
6-10	16	13%
More than 10	16	13%
BDD cases observed in the last year		
None	46	36%
1-2	54	43%
3-5	18	14%
6-10	4	3%
More than 10	5	4%
Approach to BDD patient		
Referral to psychiatrist	43	34%
Treating according to a psychiatrist's advice	5	4%
Reassurance and explanation	78	61%
Starting pharmacological therapy	1	1%
First-line choice of pharmacological therapy		
Placebo	12	30%
Anti-depressant	22	55%
Anti-psychotic	3	8%
Anti-anxiety	3	7%

Univariate and multivariate analysis 

Univariate analysis exploring the association between sociodemographic and clinical characteristics of participating dermatologists and their exposure to BDD patients or their management approach toward these patients failed to reveal any association between these characteristics and both their exposure to BDD patients and their preferred management approach (Tables 3, 4).

**Table 3:** Association between sociodemographic and work-related characteristics of dermatologist and exposure to BDD patients BDD: body dysmorphic disorder.

Characteristics	Variables	Ever encounter BDD patient (N=118)	Never encounter BDD patient (N=9)	All participants (N=127)	P-value
Age	20-30	25	4	29	0.347
	31-40	63	2	65	
	41-50	15	2	17	
	51-60	11	1	12	
	>60	4	0	4	
Gender	Male	57	3	60	0.386
	Female	61	6	67	
Nationality	Saudi	111	9	120	0.452
	Non-Saudi	7	0	7	
Clinical setting	Governmental	65	8	73	0.092
	Private	11	1	12	
	Mixed	41	0	41	
Professional position	Consultant	92	5	97	0.127
	Resident	26	4	30	
Work experience	One year	2	1	3	0.462
	1-5 years	35	3	38	
	6-10 years	42	2	44	
	11-20 years	26	2	28	
	21-50 years	13	1	14	

**Table 4:** Association between sociodemographic and work-related characteristics of dermatologists and management approach toward BDD patients BDD: body dysmorphic disorder.

Characteristics	Variables	Active management (N=49)	Passive management (reassurance and explanation) (N=78)	All participants (N=127)	P-value
Age	20-30	13	16	29	0.339
	31-40	21	44	65	
	41-50	6	11	17	
	51-60	6	6	12	
	>60	3	1	4	
Gender	Male	22	38	60	0.675
	Female	27	40	67	
Nationality	Saudi	48	72	120	0.174
	Non-Saudi	1	6	7	
Clinical setting	Governmental	26	47	73	0.486
	Private	4	8	12	
	Mixed	19	22	41	
Professional position	Consultant	36	61	97	0.541
	Resident	13	17	30	
Work experience	One year	1	2	3	0.116
	1-5 years	13	25	38	
	6-10 years	21	23	44	
	11-20 years	6	22	28	
	21-50 years	8	6	14	

## Discussion

BDD is relatively frequent but underestimated in the dermatology setting since patients usually have their perceived minor flaws “corrected” by a dermatologist or plastic surgeon rather than addressing their exaggerated preoccupation with these defects with the help of a psychiatrist. A study evaluating BDD in the psychiatry setting found that 46% of BDD sufferers had consulted a dermatologist, and 38% had actually undergone a dermatologic procedure [5].

It is difficult to obtain an accurate measure of the prevalence of BDD because the existing epidemiological studies on the subject were conducted in different clinical settings, on different patient populations, and using different diagnostic criteria [10]. Epidemiological studies suggest that the prevalence of BDD in the general population is approximately 2%, with estimates ranging from 1% to 5.3% [11-14]. However, in dermatology settings, the prevalence is significantly higher, ranging from 5% to 15% [15], with a U.S. study reporting a prevalence of 12% among dermatology patients [16]. In Turkey, Uzun et al. found that 9% of acne patients had BDD [17]. Additionally, a literature review by Koran et al. indicated a prevalence of 8.5% to 21% among dermatology patients [11].

The present study supports previous findings that BDD is commonly encountered in dermatology practice. Given that most BDD patients initially seek treatment from dermatologists rather than psychiatrists, it is crucial for dermatologists to recognize the condition and implement appropriate management strategies, including timely psychiatric referral [18,19].

A study examining dermatologic surgeons’ awareness of BDD and their ability to identify and manage these patients presenting to a dermatologic surgery service concluded that the majority of them were aware of BDD and estimated that about 13% of the patients presenting for new consultations suffered from BDD [20]. In line with that, Szepietowski et al. found that about two-thirds of the surveyed dermatologists in Poland had encountered at least one patient suffering from BDD, and more than one-fifth had managed at least one BDD patient in the last year [10]. In SA, Kattan et al. evaluated various cosmetic treatment providers’ awareness of and experience with BDD. They found that about half of these providers who were aware of the BDD diagnostic criteria had encountered at least one BDD patient within the last 12 months [21]. In the current study, more than 90% of dermatologists in SA had seen at least one BDD patient during their entire working period, and about two-thirds had seen a BDD patient in the current year. There was no association between sociodemographic and clinical characteristics of dermatologists and their exposure to BDD patients.

Physicians who encounter BDD patients acknowledge that these patients tend to be challenging to manage. They often shop among multiple skincare providers across different specialties, hiding their exaggerated preoccupation with non-existent or minor skin flaws and any related obsessive behavior. They frequently demand unnecessary therapies and pressure doctors to perform unjustified procedures, which they ultimately will not be satisfied with because they usually have unrealistic expectations. In a recent study, Crerand et al. reported that only about 4% to 7% of BDD patients are satisfied with the outcome of their procedure, and only 1% become free of their preoccupation with their defects. If anything, they tend to become even more preoccupied with the original defect or find a new one to focus on [22].

As documented in the literature, these dissatisfied patients have been known to threaten their treating physician verbally, physically, or legally [20,23]. Cultural factors and communication styles dictate the difference in the prevalence of these destructive behaviors. It is perhaps due to this emotional instability that many cosmetic professionals agree that BDD is a contraindication for any cosmetic intervention. Sarwer et al. found that about 63% of the dermatologic surgeons they surveyed considered BDD to be a contraindication for any aesthetic intervention [20]. On the other hand, only one-third of members of the American Society for Aesthetic Plastic Surgery (ASAPS) considered BDD to be a contraindication for cosmetic surgery [23]. Some authors have gone beyond this by suggesting that aesthetic intervention might be beneficial for patients with mild to moderate BDD [24].

Physicians differ in their approach to managing BDD patients. Some treat them just like any other patient, while others either discuss the disorder with them or refer them to a psychiatrist. In our study, about two-thirds of Saudi dermatologists reported that their primary approach would be to explain the nature of the condition to the patient and provide reassurance. Only 30% of those surveyed would involve a psychiatrist. This finding differs from what has been observed by other investigators. For instance, in a study that evaluated Polish dermatologists’ approach to managing BDD patients, Szepietowski et al. [10] found that two-thirds would either treat the BDD patient according to psychiatric advice or make a formal referral to a psychiatrist, whereas only 20% of those dermatologists would explain the nature of the condition and provide reassurance.

Cultural differences might underlie these different responses. The population in SA is considered conservative, and there may be a form of stigma associated with mental illness. This could make it difficult for treating physicians to disclose the diagnosis, issue a formal psychiatric referral, or prescribe psychotropic medications. In the current study, we found that most dermatologists in SA opted to avoid involving a psychiatrist in the management of their BDD patients. This finding is consistent with a previous study of Saudi cosmetic treatment providers, which found that only about one-third would involve a psychiatrist in the management of their BDD patients. In the present study, there was no association between sociodemographic and clinical characteristics of dermatologists and their management approach toward these patients.

Numerous studies have shown that selective serotonin reuptake inhibitors (SSRIs) are an effective treatment option for patients suffering from BDD. They are even considered to be the treatment of choice for patients who have suicidal ideation, with response rates ranging from 63% to 83%. Citalopram, escitalopram, fluoxetine, and fluvoxamine are among the best-studied SSRIs. In the present study, more than half (55%) of the surveyed dermatologists were aware of this medical evidence and would use antidepressants as their first-line treatment for patients suffering from BDD if they opted to actively treat these patients. On the other hand, previous studies conducted in other countries found that only 7% of the surveyed dermatologists would use antidepressants as their first-line BDD management. Additionally, Phillips et al. recommended that pharmacotherapy be coupled with psychological therapy, or “talk therapy,” which is usually conducted by a specialized therapist. Cognitive-behavioral therapy (CBT) is considered to be the psychotherapy of choice for treating BDD patients because it focuses on changing irrational, troublesome thoughts and behaviors [25]. In the present study, there was no association between sociodemographic and clinical characteristics of dermatologists and their exposure to BDD patients or their management approach toward these patients.

Although a definitive diagnosis of BDD cannot be achieved except through a well-structured psychiatric interview, recognition of this disorder is paramount for its proper treatment. Moreover, identification of this disorder is usually difficult because patients tend to hide their exaggerated preoccupation with non-existent or minor body or skin defects. In addition, these patients usually suffer from secondary anxiety or depression, which can mask the main symptoms of BDD. Therefore, using a simple self-report questionnaire, such as that proposed by Phillips, can help practicing dermatologists determine whether their patients are worried about their physical appearance, preoccupied with these thoughts, and whether these thoughts are impacting their quality of life [25]. This will ultimately increase the index of suspicion among treating physicians regarding the probability of a BDD diagnosis.

This study has several limitations that necessitate careful interpretation of the results. First, the low sample size may have reduced the precision of the obtained results. Second, the fact that this study was limited to dermatologists practicing in three major cities in SA, i.e., Riyadh, Jeddah, and Dammam, means that it might not be representative of the experiences and treatment approaches of dermatologists practicing in smaller cities. Third, the survey’s reliance on self-reporting by dermatologists may result in responses that do not accurately reflect their actual behavior when encountering BDD patients in real practice. Fourth, participating dermatologists' knowledge about BDD diagnostic criteria was not evaluated in the current study. Therefore, the magnitude of exposure to BDD cases may be overestimated or underestimated. Finally, the data obtained for this survey were based on hindsight and were therefore strongly dependent on participant recall of their patient encounters.

## Conclusions

In conclusion, we found that most of the surveyed dermatologists in SA encounter BDD patients in their daily practice. Although more than half of them were aware that early involvement of a psychiatrist in the management of BDD patients is paramount, the majority reported that their primary approach would be to explain the nature of the condition to the patient and provide reassurance. Our findings suggest that dermatologists in SA may not be sufficiently prepared to treat these patients successfully.

## Appendices

**Table 5:** Body dysmorphic disorder awareness among dermatologists in Saudi Arabia

Dermatologists’ Experience in Managing Body Dysmorphic Disorder in Saudi Arabia
Please indicate your age
20-30
31-40
41-50
51-60
Above 60
Please indicate your gender
Male
Female
Please indicate your nationality
Saudi
Non-Saudi
Please indicate the status of your institution/organization/corporate
Governmental
Private
Mixed
Please indicate your position title in your division/department
Consultant
Resident
Intern
Please indicate the length of your practice (years) in the field of dermatology
One year
1-5
6-10
11-20
21-50
How many cases of body dysmorphic disorder have you observed in your practice in your whole working period?
None
1-2
3-5
6-10
More than 10
How many cases of body dysmorphic disorder have you observed in your practice in the last five years?
None
1-2
3-5
6-10
More than 10
How many cases of body dysmorphic disorder have you observed in your practice this year?
None
1-2
3-5
6-10
More than 10
What would be your approach, if you suspected that your patient is suffering from body dysmorphic disorder?
Referral to a psychiatrist
Treatment according to a psychiatrist's advice
Reassurance and explanation
Starting pharmacological therapy
If you planned to treat the patient that you suspected suffering from body dysmorphic disorder by yourself, what would be your initial pharmacological intervention?
I will not treat this patient
Placebo
Anti-depressant
Anti-psychotic
Anti-anxiety
